# Strengthening Recruitment and Retention: Mitigation Strategies in Two Longitudinal Studies of Pregnant Women in Pakistan

**DOI:** 10.1007/s10995-024-03957-9

**Published:** 2024-06-21

**Authors:** Ilona S. Yim, Naureen Akber Ali, Aliyah Dosani, Sharifa Lalani, Neelofur Babar, Sidrah Nausheen, Shahirose Sadrudin Premji, Maternal-infant Global Health Team (MiGHT) – Collaborators in Research

**Affiliations:** 1 Department of Psychological Science, University of California, Irvine, 4201 Social and Behavioral Sciences Gateway, Irvine, CA 92697-7085 USA; 2 School of Nursing and Midwifery, Aga Khan University, Karachi, Pakistan; 3 School of Nursing and Midwifery, Faculty of Health, Community & Education, Mount Royal University, Calgary, Canada; 4 Department of Community Health Sciences and O’Brien Institute for Public Health, University of Calgary, Calgary, Canada; 5 Department of Obstetrics and Gynecology, Aga Khan University, Karachi, Pakistan; 6 School of Nursing, Faculty of Health Sciences, Queen’s University, Kingston, Canada

**Keywords:** Recruitment, Retention, Global health, LMIC, MiGHT

## Abstract

**Purpose:**

Global health researchers have a responsibility to conduct ethical research in a manner that is culturally respectful and safe. The purpose of this work is to describe our experiences with recruitment and retention in Pakistan, a low-middle-income country.

**Description:**

We draw on two studies with a combined sample of 2161 low-risk pregnant women who participated in a pilot (n = 300) and a larger (n = 1861) prospective study of psychological distress and preterm birth at one of four centers (Garden, Hyderabad, Kharadar, Karimabad) of the Aga Khan University Hospital in Karachi, Pakistan.

**Assessment:**

Challenges we encountered include economic hardship and access to healthcare; women’s position in the family; safety concerns and time commitment; misconceptions and mistrust in the research process; and concerns related to blood draws. To mitigate these challenges, we developed culturally acceptable study incentives, involved family members in the decision-making process about study participation, partnered with participants’ obstetrician-gynecologists, accommodated off site study visits, combined research visits with regular prenatal care visits, and modified research participation related to blood draws for some women.

**Conclusion:**

Implementation of these mitigation strategies improved recruitment and retention success, and we are confident that the solutions presented will support future scientists in addressing sociocultural challenges while embarking on collaborative research projects in Pakistan and other low-middle-income countries.

## Significance

**What is Already Known on this Subject?:**

Recruitment and retention of pregnant women is challenging in any research context, for a range of socioecological reasons, including societal, community-level and individual factors. These issues can be more emphasized or play out in unique ways in studies conducted in low-middle-income countries.

**What this Study Adds?:**

This study describes recruitment and retention challenges we encountered during our research in Pakistan. For each of these challenges, which may result in non-compliance if not addressed, the development and implementation of culturally safe and successful mitigation strategies is described. The knowledge gleaned may support future global health research teams setting out to conduct research in Pakistan, and perhaps other low-middle-income countries.

## Purpose

Conducting research in a low- and middle-income country (LMIC) context poses unique challenges, some of which are distinct from those typically encountered in high income countries. An awareness of possible challenges and culturally safe approaches to addressing them should be part of the repertoire of any global health research team (Lahey, 2013). The purpose of this article is to report on recruitment and retention challenges encountered in two prospective studies of psychological distress and preterm birth among pregnant women in Pakistan, and to describe the mitigation strategies implemented to successfully conduct this research. In doing so, we will emphasize the local context in which our study was embedded.

## Description

For this report, we draw on two studies, a pilot project of 300 (e.g., Lalani et al., 2021; Premji et al., 2020) and a follow up study of 1861 (Lalani et al., 2023) low-risk pregnant women receiving antenatal care and delivering at one of four centers (Garden, Hyderabad, Kharadar, Karimabad) of the Aga Khan University Hospital in Karachi, Pakistan. Briefly, the overarching goal of both studies was to prospectively assess the link between perinatal distress and preterm birth in an LMIC context. Pregnant women were first assessed between 10 and 19 or between 12 and 19 weeks’ gestational age, depending on the study, and then followed up at 22 to 29 weeks’ gestational age. Birth outcomes were then assessed at the time of delivery. Ethics approval for Study 1 was obtained from the Ethics Research Committee, Aga Khan University Hospital (ID: 3564-SON-ERC-15) and the Conjoint Health Research Ethics Board, University of Calgary (ID: REB15-2372). Study 2 was approved by the National Bioethics Committee Pakistan [No.4-87/NBC-286-Y2]; the Aga Khan University Ethics Review Committee [5003-SON-ERC-17], Karachi, Pakistan; Mount Royal University Human Research Ethics Board [File ID#101116]; the University of Calgary Conjoint Health Research Ethics Board [REB17-1148_REN5]; York University Office of Research Ethics [2018–184]; and Queen’s University Health Sciences & Affiliated Teaching Hospitals Research Ethics Board [NURS-566-23].

Conducting research in an LMIC context poses challenges that differ from those encountered in high income countries, and these challenges are unique to the sociocultural context in which the research occurs. Adding layers of complexity to our recruitment and retention experience in an LMIC context are the involvement of pregnant women as research subjects and the longitudinal nature of our study. Conducting longitudinal studies with pregnant women is challenging in any context for a range of socioecological reasons, including institutional, legal and higher-level factors (e.g., federal guidelines, liability issues), community and social level factors (e.g., clinic and study accessibility, spouse/partner influences, and household dynamics), and individual-level factors (e.g., time to participate, health-related issues, and fear; Frew et al., 2014). All of these factors apply in LMICs, although the impact of some of them is significantly enhanced. We will not reiterate all relevant issues here, but emphasize those that were enhanced or unique to our study in Pakistan. These included challenges related to economic hardship and access to healthcare; women’s position in the family; safety concerns and time commitment; misconceptions and mistrust in the research process; and concerns related to blood draws. For each set of challenges, we describe mitigation strategies that were successfully employed.

## Assessment

### Economic Hardship and Access to Healthcare

Women in LMIC face more economic hardship than women in high income countries. They also have less access to health care, with disparities in the quality, geographic accessibility, availability, and acceptability of health care services (Peters et al., 2008). Many women we approached for study participation experienced unemployment, economic hardship, and poverty in an environment characterized by a high cost of living. Women did not typically seek medical care, even in times of extreme need, as costs are out of pocket and health care is often unaffordable. These circumstances affect pregnancy, because women frequently enter pregnancy in an anemic state and prefer home deliveries. Thus, pregnancy often results in severe morbidity and mortality.

Against this socioeconomic backdrop, identifying an appropriate incentive for study participation poses unique ethical challenges, as even a small monetary incentive for research participation may be coercive. This issue was compounded by the fact that not all women in our study had control over family finances and may not have had a say in how money received in exchange for study participation ought to be used. Providing free health care as a study incentive was similarly fraught with problems, because women may have joined the study only to receive that benefit. We also did not offer free transportation to the study site, because of a concern that free transportation to the hospital may become an expectation, even for regular antenatal visits, and that some women might forgo antenatal visits for that reason.

To mitigate these issues, the research team instead covered 50% of the cost of follow up care for all participants. This approach was successful, was perceived to be fair by participants, and, in all cases, posed a direct benefit to the participating women. Moreover, because this was a study of psychological distress and preterm birth, and non-psychotic perinatal mental health disorders occur at substantially higher rates in LMIC compared to high income countries (Fisher et al., 2012), we also offered free services with a female psychiatrist to women who scored high on scales assessing depressive symptoms or anxiety. In our study, 245 women were offered this opportunity, and 19 women took advantage of this service. All women received a list of mental health resources they could access.

### Women’s Position in the Family Unit

Our study occurred within a cultural context where many women may be in a subservient position inside a patriarchal family structure, with husbands and mothers-in-law holding central and fundamental decision-making power (Neelotpol et al., 2016). In this circumstance, a lack of support from a spouse or mother-in-law has been shown to be a strong barrier for pregnant women to participate in research (Neelotpol et al., 2016; Rohra et al., 2009). In our study, about one in twenty women (5.2%, n = 97) refused participation after discussing research details with their family members. The reason behind the refusal by the family members, most frequently the husband, was often an extreme worry about the health of the mother and fetus. Sometimes, women did not understand the aims and purpose of the research well, which made it difficult for them to communicate this to their family members, resulting in the family’s decision not to participate. We also noticed that women and their family members were hesitant to ask questions about the research, and would rather decline participation than ask for clarification. Thus, family members’ attitudes or opinions about a research study can discourage or prevent pregnant women’s recruitment and retention.

To mitigate this challenge, we involved family members along with pregnant women in the process of recruitment, whenever questions and concerns were brought up to the study team, or when study-related guidance was sought out from the woman’s gynecologist. By educating and counseling all parties about research and the intricacies of this study, we gained family members’ trust and support, and made sure that all decision makers had relevant information in its entirety. With the implementation of these strategies, our recruitment and retention rates significantly improved.

### Safety Concerns and Time Commitment

The day-to-day living situation of our participants was characterized by political insecurity and unrest in the country. Many participants cited safety concerns related to traveling to the study cite, as well as transportation issues more generally, as a hurdle to participation and timely follow-up, as many women lived far away from study sites. Moreover, it was not considered culturally appropriate for some women in these areas to travel alone, and women had to be accompanied by a male family member to bring them to the hospital for their medical care and study participation. Women were also concerned that they would have to invest a significant amount of time in the study that they could otherwise spend taking care of their family. Logistical issues and lack of time were challenges that have previously been identified as significant barriers in recruiting and retaining participants in studies in South Asia (Quay et al., 2017).

We mitigated these concerns by scheduling study visits alongside the prenatal health care visits, which circumvented the need for additional research-related travel. When this was not possible or feasible, we offered the option of research staff and phlebotomists traveling to women’s homes to complete measures and collect the blood sample. For women who did not consent to a home visit, we put in place a strong communication system with nearby [Aga Khan University Hospital]﻿ laboratory collection units and administrative personnel so that pregnant women could visit a nearby site to provide a blood sample. In those cases, research staff completed questionnaires with women over the phone. To mitigate the concern related to time spent on research, the time for study participation was kept short, with study visits not exceeding 30 min. Most women participated while waiting to see their doctor for regular antenatal visits. Therefore, for the most part, research participation did not, or at least not substantively, add extra time to pregnant women’s schedules.

### Misconceptions and Mistrust in the Research Process

Women approached for study participation often had misconceptions about research and sometimes mistrusted the research process. These concerns are not atypical for research studies conducted in low-resource settings, where socioeconomic disadvantage, high illiteracy rates, a general lack of access to healthcare, and low participation in research studies combine to form a set of circumstances where exploitation in research is more likely to occur (Colom & Rohloff, 2018; Schroeder et al., 2019).

Many women in our study initially expressed beliefs or had misconceptions that taking part in research may harm their pregnancy or lead to adverse pregnancy outcomes, contributing to their reluctance to participate. For example, women expressed concerns that participation in the study and giving blood samples could lead to anemia, preterm labor, miscarriage, fetal growth restriction or intrauterine death. Similar concerns about potential harm to the pregnancy have been reasons for non-participation in previous studies, including those in LMIC (e.g., Chansamouth et al., 2017). In addition, some participants were concerned that giving consent or providing their signature or thumbprints on the consent form might result in losing their property, their land, their house, or their essential belongings. These concerns have in common that they are based in mistrust, which is a challenge often encountered in LMIC research (Colom & Rohloff, 2018).

To mitigate these concerns, building trust with research participants and their families was critical. Our project research staff, the majority of whom belonged to the same community as the research participants, responded respectfully, politely, and promptly to women’s concerns during recruitment and throughout the study. We closely collaborated with women’s obstetrician-gynecologists who acknowledged women’s concerns and fears, provided advice and clarification related to research, and reassured women that research participation is an option they can consider. By involving a trusted member of the pregnant woman’s health care network, we were able to increase women’s trust in the research team as a whole and to significantly increase recruitment success. Engaging in this conversation provided support and education to pregnant women, lessened their hesitation and enhanced participation.

Another way to address misconceptions about research is to use language that is appropriate to the sociocultural context in which a study is embedded. Given the wide range of educational attainment and cultural diversity across the four health care centers from which we recruited, we created all study materials, including the consent form, using simple language without medical jargon to explain research methods and procedures. Consent forms and study materials were made available in the three predominant local languages: Urdu, Sindhi, and English, and multilingual research assistants were on site to answer questions in women’s preferred language. This assisted women to read and comprehend the consent form and study materials, and made it easier for them to ask relevant questions. If women were unable to read the consent form, it was read to them and questions were addressed. It has been previously suggested that research participants’ understanding may be improved if adequate time is taken in reading the consent form, and topics or procedures are clarified using simple and appropriate language (Kadam, 2017).

### Concerns about Blood Collection

Our study protocol involved the collection of blood, and some women and their families shared with us that they were concerned about needle pricks, the possibility that a blood draw during pregnancy could contribute to anemia, and that blood draws may be in conflict with their religious beliefs, especially while fasting during Ramadan. Some participants thought that the blood draw might break their fast, or that weakness and vertigo might result from a blood draw while fasting. Similar concerns have been previously reported (Peiffer-Smadja et al., 2017). Some participants worried that the study team might use the blood sample to conduct tests other than those listed on the consent form, including for example HIV tests, and that the results of these tests might lead to significant adverse consequences to their lives.

To mitigate the concerns regarding repeated blood draws, we scheduled blood sampling to coincide with routine blood draws whenever possible, minimizing additional pricks. Concerns about using their blood outside the scope of this study were addressed by the obstetrician-gynecologist during antenatal visits. These physicians independently explained the process, reassured participants, and addressed any unique health concerns related to study procedures. This approach went a long way in reducing misconceptions, and in assuring that women and their families made well-informed decisions about study participation. We also learned that it was necessary to accommodate study participation without blood sampling for women who, after these explanations, remained unwilling to provide a blood sample. In our study, 1.3% of women were initially recruited with the understanding that they would not provide a blood sample. As pregnancy progressed, maternal concerns and fear about harm to the pregnancy increased, and at follow up, 10 weeks further into their pregnancy, 15.7% of participants continued participation without blood sampling.

## Conclusion

Recruitment and retention of pregnant women into prospective, longitudinal research studies is always challenging, but more so in LMICs. Crucial challenges encountered were finding appropriate compensation due to poverty and low access to health care; navigating a sociocultural setting where women are not the sole decision makers of their own research participation; and navigating an atmosphere of mistrust and misconception about the research process, in particular as it related to obtaining blood samples. For each of these challenges, we implemented culturally sensitive mitigation strategies, which improved recruitment and retention. First, providing 50% of follow up health care cost and mental health support to women who needed and wanted it, was perceived as fair and directly benefitted the participating women. Second, involving all family decision makers throughout the research process was critical because many women were not empowered to be sole decision makers. Finally, involving trusted health care providers, who could provide reassurance and explain the research to families, was critical. Allowing modified participation protocols (e.g., participation without blood sampling) further contributed to our recruitment and retention success when women remained concerned about this aspect of their participation. In sum, these mitigation strategies were instrumental in improving recruitment and retention and played a pivotal role in successfully conducting this research. We are hopeful that this report will provide guidance to other maternal and child health researchers in Pakistan and perhaps in other LMICs in formulating strategies to improve recruitment and retention rates.

## Data Availability

No original data analyses are presented in this manuscript. The data underlying these two studies contain sensitive participant information and cannot be shared publicly.

## References

[CR1] Chansamouth, V., McGready, R., Chommanam, D., Homsombath, S., Mayxay, M., & Newton, P. N. (2017). Enrolling pregnant women in research: Ethical challenges encountered in Lao PDR (Laos). *Reproductive Health,**14*(Suppl 3), 167. 10.1186/s12978-017-0428-9 29297369 10.1186/s12978-017-0428-9 PMC5751390

[CR2] Colom, M., & Rohloff, P. (2018). Cultural considerations for informed consent in paediatric research in low/middle-income countries: A scoping review. *BMJ Paediatrics Open,**2*(1), e000298. 10.1136/bmjpo-2018-000298 30613801 10.1136/bmjpo-2018-000298 PMC6307601

[CR3] Fisher, J., Cabral de Mello, M., Patel, V., Rahman, A., Tran, T., Holton, S., & Holmes, W. (2012). Prevalence and determinants of common perinatal mental disorders in women in low- and lower-middle-income countries: A systematic review. *Bulletin of the World Health Organization,**90*(2), 139g–149g. 10.2471/blt.11.091850 22423165 10.2471/BLT.11.091850 PMC3302553

[CR4] Frew, P. M., Saint-Victor, D. S., Isaacs, M. B., Kim, S., Swamy, G. K., Sheffield, J. S., Edwards, K. M., Villafana, T., Kamagate, O., & Ault, K. (2014). Recruitment and retention of pregnant women into clinical research trials: An overview of challenges, facilitators, and best practices. *Clinical Infectious Diseases,**59 Suppl 7*(Suppl 7), S400–S407. 10.1093/cid/ciu726 25425718 10.1093/cid/ciu726 PMC4303058

[CR6] Kadam, R. A. (2017). Informed consent process: A step further towards making it meaningful! *Perspectives in Clinical Research,**8*(3), 107–112. 10.4103/picr.PICR_147_16 28828304 10.4103/picr.PICR_147_16 PMC5543760

[CR70] Lalani, S., Dosani, A., Forcheh, N., Premji, S. S., Siddiqui, S., Shaikh, K., Ayesha M., Yim, I. S. (2021). Perceived stress may mediate the relationship between antenatal depressive symptoms and preterm birth: A pilot observational cohort study. *PLoS One*, *16*(5), e0250982. 10.1371/journal.pone.0250982 33945579 10.1371/journal.pone.0250982 PMC8096039

[CR72] Lalani, S., Premji, S. S., Shaikh, K., Sulaiman, S., Yim, I. S., Forcheh, N., Babar, N., Nausheen, S., Letourneau, N., Maternal-infant Global Health Team Collaborators in, R. (2023). Individual and collective contribution of antenatal psychosocial distress conditions and preterm birth in Pakistani women. *PLoS One*, *18*(3), e0282582–e0282582. 10.1371/journal.pone.0282582 36996124 10.1371/journal.pone.0282582 PMC10062634

[CR7] Lahey, T. (2013). The ethics of clinical research in low- and middle-income countries. *Handbook of Clinical Neurology,**118*, 301–313. 10.1016/b978-0-444-53501-6.00025-1 24182387 10.1016/B978-0-444-53501-6.00025-1

[CR8] Neelotpol, S., Hay, A. W., Jolly, A. J., & Woolridge, M. W. (2016). Challenges in collecting clinical samples for research from pregnant women of South Asian origin: Evidence from a UK study. *British Medical Journal Open,**6*(8), e010554. 10.1136/bmjopen-2015-010554 10.1136/bmjopen-2015-010554 PMC5013462 27580825

[CR9] Peiffer-Smadja, N., Ouedraogo, R., D’Ortenzio, E., Cissé, P. N., Zeggani, Z., Beavogui, A. H., Faye, S. L., Le Marcis, F., Yazdanpanah, Y., & Nguyen, V. K. (2017). Vaccination and blood sampling acceptability during Ramadan fasting month: A cross-sectional study in Conakry, Guinea. *Vaccine,**35*(19), 2569–2574. 10.1016/j.vaccine.2017.03.068 28385606 10.1016/j.vaccine.2017.03.068

[CR10] Peters, D. H., Garg, A., Bloom, G., Walker, D. G., Brieger, W. R., & Rahman, M. H. (2008). Poverty and access to health care in developing countries. *Annals of the New York Academy of Sciences,**1136*, 161–171. 10.1196/annals.1425.011 17954679 10.1196/annals.1425.011

[CR100] Premji, S. S., Lalani, S., Shaikh, K., Mian, A., Forcheh, N., Dosani, A., Letourneau, N., Yim, I. S. Bhamani, S. S. Maternal-Infant Global Health Team-Collaborators In Research, M. (2020). Comorbid anxiety and depression among pregnant Pakistani women: higher rates, different vulnerability characteristics, and the role of perceived stress. *Int J Environ Res Public Health*, *17*(19). 10.3390/ijerph17197295 10.3390/ijerph17197295 PMC7579342 33036215

[CR11] Quay, T. A., Frimer, L., Janssen, P. A., & Lamers, Y. (2017). Barriers and facilitators to recruitment of South Asians to health research: A scoping review. *British Medical Journal Open,**7*(5), e014889. 10.1136/bmjopen-2016-014889 10.1136/bmjopen-2016-014889 PMC5541387 28576896

[CR12] Rohra, D. K., Khan, N. B., Azam, S. I., Sikandar, R., Zuberi, H. S., Zeb, A., Qureishi, R. N., & Hasan, R. (2009). Reasons of refusal and drop out in a follow up study involving primigravidae in Pakistan. *Acta Obstetricia et Gynecologica Scandinavica,**88*(2), 178–182. 10.1080/00016340802657561 19107618 10.1080/00016340802657561

[CR13] Schroeder, D., Chatfield, K., Singh, M., Chennells, R., & Herissone-Kelly, P. (2019). Exploitation risks in collaborative international research. In *Equitable research partnerships: A global code of conduct to counter ethics dumping* (pp. 37–50). Springer International Publishing.

